# Gene-variant specific effects of plasma amyloid-β levels in Swedish autosomal dominant Alzheimer disease

**DOI:** 10.1186/s13195-024-01574-w

**Published:** 2024-09-25

**Authors:** Charlotte Johansson, Steinunn Thordardottir, José Laffita-Mesa, Josef Pannee, Elena Rodriguez-Vieitez, Henrik Zetterberg, Kaj Blennow, Caroline Graff

**Affiliations:** 1 Department NVS, Division of Neurogeriatrics, Center for Alzheimer Research, Karolinska Institutet, Visionsgatan 4, Bioclinicum, Solna, J10:20, 171 64 Sweden; 2 Theme Inflammation and Aging, Karolinska University Hospital, Stockholm, Sweden; 3 Clinical Neurochemistry Laboratory, Sahlgrenska University Hospital, Mölndal, Sweden; 4 Department of Psychiatry and Neurochemistry, Institute of Neuroscience and Physiology, Sahlgrenska Academy, University of Gothenburg, Mölndal, Sweden; 5 Department of Neurodegenerative Disease, UCL Institute of Neurology, London, UK; 6 UK Dementia Research Institute at UCL, London, UK; 7 Pitié-Salpêtrière Hospital, Paris Brain Institute, ICM, Sorbonne University, Paris, France; 8 Neurodegenerative Disorder Research Center, Division of Life Sciences and Medicine, Department of Neurology, Institute on Aging and Brain Disorders, University of Science and Technology of China and First Affiliated Hospital of USTC, Hefei, P.R. China

**Keywords:** Alzheimer disease, Autosomal dominant, Amyloid-β, Plasma biomarkers, APP

## Abstract

**Background:**

Several blood-based biomarkers offer the opportunity of in vivo detection of brain pathology and neurodegeneration in Alzheimer disease with high specificity and sensitivity, but the performance of amyloid-β (Aβ) measurements remains under evaluation. Autosomal dominant Alzheimer disease (ADAD) with mutations in *PSEN1*, *PSEN2* and *APP* can be studied as a model for sporadic Alzheimer disease. However, clarifying the genetic effects on the Aβ-levels in different matrices such as cerebrospinal fluid or plasma is crucial for generalizability and utility of data. We aimed to explore plasma Aβ concentrations over the Alzheimer disease continuum in a longitudinal cohort of genetic Alzheimer disease.

**Methods:**

92 plasma samples were collected from at-risk individuals (*n* = 47) in a Swedish cohort of ADAD, including 18 mutation carriers (13 *APP*swe (p.KM670/671NL) MC), 5 *PSEN1* (p.H163Y) MC) and 29 non-carriers (NC) as the reference group. Concentrations of Aβ1–38, Aβ1–40 and Aβ1–42 were analyzed in plasma using immunoprecipitation coupled to tandem liquid chromatography mass spectrometry (IP-LC-MS/MS). Cross-sectional and repeated-measures data analyses were investigated family-wise, applying non-parametric tests as well as mixed-effects models.

**Results:**

Cross-sectional analysis at baseline showed more than a 3-fold increase in all plasma Aβ peptides in *APP*swe MC, regardless of clinical status, compared to controls (*p* < 0.01). *PSEN1* (p.H163Y) presymptomatic MC had a decrease of plasma Aβ1–38 compared to controls (*p* < 0.05). There was no difference in Aβ1–42/1–40 ratio between *APP*swe MC (PMC and SMC), *PSEN1* MC (PMC) and controls at baseline. Notably, both cross-sectional data and repeated-measures analysis suggested that *APP*swe MC have a stable Aβ1–42/1–40 ratio with increasing age, in contrast to the decrease seen with aging in both controls and *PSEN1* (p.H163Y) MC.

**Conclusion:**

These data show very strong mutation-specific effects on Aβ profiles in blood, most likely due to a ubiquitous production outside of the CNS. Hence, analyses in an unselected clinical setting might unintentionally disclose genetic status. Furthermore, our findings suggest that the Aβ ratio might be a poor indicator of brain Aβ pathology in selected genetic cases. The very small sample size is a limitation that needs to be considered but reflects the scarcity of longitudinal in vivo data from genetic cohorts.

**Supplementary Information:**

The online version contains supplementary material available at 10.1186/s13195-024-01574-w.

## Introduction

Autosomal dominantly inherited Alzheimer disease (ADAD) has many similarities to and can inform on the nature of pathological processes also in the more common sporadic forms of Alzheimer disease (AD). Indeed, the genetic underpinnings of ADAD contributed to the identification of aberrant processing of amyloid precursor protein as an initiating event of AD pathology [1]. Pathogenic variants in the amyloid precursor protein (*APP*), presenilin 1 (*PSEN1*) or presenilin 2 (*PSEN2*) genes cause an early onset AD phenotype, usually with conventional AD proteinopathy of aggregated amyloid beta (Aβ) and hyperphosphorylated tau (P-tau) in deposits of neuritic plaques and neurofibrillary tangles [2]. ADAD pathogenic variants typically cause an overall increase in the production of the APP cleavage products Aβ40 and Aβ42 or a relative increase in Aβ42 peptides, which are known to have more amyloidogenic properties [3–6]. Furthermore, changes in the intrinsic properties of Aβ have been reported, as in the case with the *Arctic APP* (*APP*arc, (p.E693G)) and the *Uppsala APP* (*APP* p.Δ690–695) mutations, where the location of the mutations within the Aβ sequence also results in a more fibrillogenic peptide product [7, 8]. Understanding such effects and variations in the pathogenic substrate is crucial for the interpretation and generalizability of ADAD research data.

Studies of healthy individuals at-risk for ADAD provide a unique representation of AD pathology as measured by fluid and imaging biomarkers also in the preclinical phase of the AD continuum. One of the first detectable abnormalities in ADAD is the decrease of Aβ42 concentrations in cerebrospinal fluid (CSF) in mutation carriers (MC) compared to non-carrier (NC) controls. This occurs more than 20 years before symptom onset [9, 10], sometimes from an early high concentration in young age [11]. Similar to sporadic AD, the decrease in CSF Aβ42 is followed later by increased 11 C Pittsburgh compound-B (PiB) binding in positron emission tomography (PET) and evidence of tau pathology and neurodegeneration [9, 12], aligning with the order of the A/T/N classification concept [13] and the amyloid cascade hypothesis [1]. The inverse correlation between soluble Aβ42 in CSF and PiB-PET retention is considered to reflect successive deposition of Aβ in insoluble neuritic amyloid plaques [14].

Early evaluation of blood-based Aβ biomarkers of CNS AD pathology showed partly inconsistent results, suggesting both decreased and increased levels of plasma Aβ42 and Aβ42/40 ratio in sporadic AD compared to controls [15, 16]. Since then, mass spectrometry-based methodology has confirmed that plasma Aβ42 and the Aβ42/40 ratio decrease in individuals with an increased deposition of Aβ in brain as detected by PET [17], confirming a weak positive correlation and alignment with CSF Aβ concentrations [18]. Furthermore, plasma Aβ levels have been repeatedly associated with abnormal CSF Aβ42/40 and Aβ PET status in several studies [18–21]. However, recent multicenter studies, comparing different immunological and mass spectrometric methods, showed only weak correlations for plasma Aβ42 concentrations and moderate correlations for Aβ40 between assays, which contrasted to the very high correlations between different CSF assays [22, 23]. Hence, the robustness and utility of plasma Aβ for clinical trials and clinical practice remain unclear [24, 25].

We aimed to explore plasma Aβ concentrations cross-sectionally and in an exploratory repeated-measures analysis over the ADAD continuum in a longitudinal cohort from Sweden. Additionally, associations between plasma Aβ and CSF concentrations of core AD biomarkers were assessed.

## Methods

### Study design and participants

Affected and at-risk adult relatives from the Swedish familial Alzheimer disease study contributed with clinical data, CSF and blood samples, as described previously [26]. Symptomatic mutation carriers (SMC) and presymptomatic mutation carriers (PMC) from two ADAD families (*APP*swe (p.KM670/671NL) and *PSEN1* (p.H163Y)) as well as non-carriers (NC) from three families (*APP*swe, *APP*arc (p.E693G) and *PSEN1* (p.H163Y)) were included in the study. Sampling was performed during the years 1994 to 2018. Participants and study personnel were only informed of mutation status if the participant had performed a clinical presymptomatic genetic test and disclosed the results. The mean age at symptom onset was 52 ± 6 years (mean ± SD) in *PSEN1* (p.H163Y) mutation carriers (based on 12 affected individuals) and 54 ± 5 years in *APP*swe mutation carriers (based on 24 affected individuals), estimated from all available information from each family.

All plasma and CSF biomarkers were analyzed, as described below, at the Clinical Neurochemistry Laboratory at the Sahlgrenska University Hospital, Mölndal Sweden.

### Blood sample collection

Venipuncture was performed in non-fasting subjects during daytime, using either sodium heparin (before year 2015) or ethylenediaminetetraacetic acid (EDTA, after year 2015) anticoagulant additives. In total, 10 of the 92 blood samples were collected in EDTA tubes, two from *APP*swe MC (one at baseline), three from *PSEN1* MC (none at baseline) and five samples from NC controls (one at baseline). Within the hour, samples were centrifuged for 10 min at 2200 g at + 20^0^C. The supernatant plasma was aliquoted into 1mL polypropylene tubes and frozen at -80^0^C. Samples were thawed on ice and re-aliquoted before re-freezing and transportation to the laboratory at the Sahlgrenska University Hospital, where samples were thawed again for the Aβ mass spectrometry.

### Mass spectrometry analysis of Aβ peptides

Detection of plasma Aβ1–38, Aβ1–40 and Aβ1–42 was performed by immunoprecipitation coupled to tandem liquid chromatography mass spectrometry (IP-LC-MS/MS), using an in-house protocol, as described previously [21, 27]. In short, calibrators were prepared using recombinant Aβ1–38, Aβ1–40 and Aβ1–42 (rPeptide) added to 8% bovine serum albumin in phosphate-buffered saline. Recombinant ^15^N labeled Aβ1–38, Aβ1–40 and Aβ1–42 were used as internal standards (IS), added to samples and calibrators prior to sample preparation. Immunoprecipitation with anti-β-Amyloid 17–24 (4G8) and anti-β-Amyloid 1–16 (6E10) antibodies (both Biolegend^®^) coupled to Dynabeads™ M-280 Sheep Anti-Mouse IgG magnetic beads (Thermofisher, Waltham, MA, USA) was performed using a KingFisher™ Flex Purification System (Thermofisher, Waltham, MA, USA). A Dionex Ultimate LC-system and a Thermo Scientific Q Exactive quadrupole-Orbitrap hybrid mass spectrometer was used for LC-MS/MS. Chromatographic separation was achieved using basic mobile phases and a reversed-phase monolith column at a flow rate of 0.3 mL/min. The mass spectrometer operated in parallel reaction monitoring (PRM) mode and was set to isolate the 4 + charge state precursors of the Aβ peptides. Product ions (14–15 depending on peptide) specific for each precursor were selected and summed to calculate the chromatographic areas for each peptide and its corresponding IS. The area ratio of the analyte to the internal standard in unknown samples and calibrators was used for quantification. In summary, this was a targeted MS method set to detect only plasma Aβ1–38, Aβ1–40 and Aβ1–42, omitting other peptides.

### CSF collection and analysis

Collection of CSF samples was performed during the years 1993 to 2015. CSF was collected into polypropylene tubes and immediately centrifuged at 3000×g at + 4 °C for 10 min. The supernatant was pipetted off, aliquoted into polypropylene cryotubes and stored at − 80 °C [10, 28]. The assays for measurements of CSF Aβ peptides, P-tau181 and T-tau concentrations were designed and analyzed twice each as previously described [28, 29] and duplicate results were averaged before introduced into the statistical analyses. Measurements of CSF Aβ peptides (Aβ38, Aβ40 and Aβ42) were performed using electrochemiluminescence technology, with the MS6000 Human Abeta 3-Plex Ultra-Sensitive Kit (detection antibody 6E10), as recommended by the manufacturer (Meso Scale Discovery, Gaithersburg, Maryland, USA). CSF P-tau181 concentrations were measured by the INNOTEST^®^ phospho-tau 181P ELISA (Fujirebio Europe, Ghent, Belgium) [30] and T-tau by using a sandwich ELISA (INNOTEST TAU-Ag, Fujirebio Europe, Ghent, Belgium), designed to measure all tau isoforms regardless of phosphorylation status [31, 32]. All analyses were performed at the Clinical Neurochemistry Laboratory at the Sahlgrenska University Hospital, Mölndal, Sweden by certified laboratory assistants, blind to clinical data.

### *APOE* genotyping

The *APOE* genotyping was performed for SNPs rs7412 and rs429358 using Taqman^®^ SNP Genotyping Assays (Thermofisher, Waltham, MA, USA) according to manufacturer’s protocol. The amplified products were run on 7500 fast Real-Time PCR Systems (Thermofisher, Waltham, MA, USA). Participants who were carriers of one or two alleles of ɛ4 were categorized as *APOE* ɛ4 positive.

### *APP* and *PSEN1* genotyping

Exon 16 and 17 in the *APP* gene and exon 6 in the *PSEN1* gene were re-sequenced and screened for the *APP*swe [33], the *APP*arc [7] and the *PSEN1* (p.H163Y) mutations [34]. AmpliTaq Gold^®^ 360 PCR Master Mix (Thermofisher, Waltham, MA, USA) was used for DNA amplification. Primer sequences and PCR conditions are available upon request. Sanger sequencing was performed using BigDye™ Terminator v3.1 Cycle Sequencing Kit (Thermofisher, Waltham, MA, USA) in both forward and reverse directions and analyzed using ABI3500 Genetic Analyzer (Thermofisher, Waltham, MA, USA).

### Statistical analysis

Statistical analyses were performed separately for the *APP*swe and *PSEN1* mutations due to known strong mutation-specific effects on Aβ processing in *APP*swe [5, 35, 36]. Non-carrier controls from the Swedish familial Alzheimer disease study (*APP*swe, *APP*arc and *PSEN1* (p.H163Y)) were used together as reference group. Plasma biomarker results were normally distributed in MC family-wise and in the pooled NC respectively, except for skewed Aβ1–40 concentrations in NC. Quality control indicated inconsistent plasma peptide concentrations in part of the mass spectrometry experiments due to technical issues (Suppl. Figure 1). In total 73 plasma samples were excluded from further statistical analyses, as explained in Suppl. Figure 1.

Descriptive statistical analyses were used to compare PMC, SMC and NC controls. Unpaired *t*-tests and Mann-Whitney *U* or Kruskal-Wallis tests were applied for normally distributed and skewed data, respectively. Fisher’s exact test was applied in descriptive analysis of categorical variables. Spearman correlations were used to analyze association between plasma (Aβ1–38, Aβ1–40, Aβ1–42 and Aβ1–42/1–4 ratio) and CSF biomarker (Aβ38, Aβ40, Aβ42, Aβ42/40 ratio, T-tau and P-tau181) concentrations in a subset of matching plasma and CSF samples collected at the same date. P-values < 0.05 were considered significant and always calculated from 2-sided tests. False discovery rate (FDR) correction was applied to adjust for multiple comparisons, with Q set to 5% [37].

Mixed-effects models were applied to assess the association of repeated-measurements of plasma Aβ peptide concentrations with mutation status (MC or NC) and age. Analyses included age, mutation status (MC and NC) and the mutation status-by-age interaction (mutation status*age) as fixed-effects predictors. A random intercept at the individual level was included to account for within-subject correlations. Age was centered to mean age at onset in the corresponding family (54 years for comparison of *APP*swe MC vs. NC and 52 years for comparison of *PSEN1* (p.H163Y) MC vs. NC). Also, all models were adjusted for sex and *APOE* ɛ4 + status (positive or negative). Sensitivity analyses models including quadratic (age^2 and mutation status*age^2) or cubic terms (age^3 and mutation status*age^3) did not suggest a curvilinear relationship between any Aβ concentrations and age, neither did inclusion of these predictors improve goodness-of-fit as estimated by the Akaike information criterion. Restricted maximum likelihood estimation and Satterthwaite approximations for degrees of freedom were applied [38, 39] due to small sample size.

Statistical calculations were performed using SPSS 27.0 (IBM Corporation, Armonk, NY, USA), R (R version 4.3.2, the R Foundation for Statistical Computing Platform) and R Studio software (RStudio Team; version 2023.12.1.402). The Lme4 package was used for mixed-effects models.

## Results

### Sample cohort and demographics

Cross-sectional analysis included samples from 47 individuals at baseline, whereof there were 13 *APP*swe MC samples (10 PMC and 3 SMC), 5 *PSEN1* MC samples (5 PMC) and 29 NC samples. The repeated-measures analysis included 92 samples from the 47 individuals, whereof there were 23 *APP*swe MC samples (17 PMC and 6 SMC), 20 *PSEN1* MC samples (17 PMC and 3 SMC) and 49 NC samples (Suppl. Figure 1). The total mean number of visits was 2.0 ± 1.5SD (range 1 to 8) and mean follow-up time in years was 6.2 ± 8.2SD (range 0 to 23).

Demographic and clinical characteristics of the cohort are displayed in Table 1. There were no statistically significant differences in the distributions of age, sex and *APOE* ɛ4 status between *APP*swe and *PSEN1* families, or when comparing among the SMC, PMC and NC groups. Clinical Dementia Rating Scale (CDR) was 0 in all groups, except for SMC that had a median CDR of 2.5 (Table 1).

**Table 1:** Demographics and plasma Aβ isoforms, baseline

	APPswe		PSEN1	All
	PMC	SMC	PMC	NC
	(*n* = 10)	(*n* = 3)	(*n* = 5)	(*n* = 29)
Age, y	39 (28–51)	55 (55–66)	30 (27–43)	42 (20–83)
Sex, F:M (%)	5:5 (50:50)	0:3 (0:100)	0:5 (0:100)	9:20 (31:69)
APOE ɛ4+, n (%)	7 (70)	1 (33)	4 (80)	13 (45)
CDR	0 (0)	2.5 (2–3)*	0 (0)	0 (0)
MMSE	30 (30)	NA	30 (27–30)	29 (27–30)
Aβ1–38 (pg/mL)	65.0 (48.7–80.7)***	64.6 (38.8–81.6)**	13.9 (6.6–17.6)*	17.9 (7.4–30.0)
Aβ1–40 (pg/mL)	833 (556–955)***	901 (782–949)**	229 (209–246)	251 (187–401)
Aβ1–42 (pg/mL)	87.5 (71.8-112.2)***	88.4 (84.6-110.8)**	29.1 (24.0-31.2)	27.4 (18.7–40.5)
Aβ1–42/1–40	0.108 (0.087–0.129)	0.113 (0.094–0.117)	0.126 (0.115–0.136)	0.116 (0.062–0.146)

Age, CDR, MMSE scores and all plasma biomarker values are expressed in median (range). Kruskal Wallis test, Mann-Whitney U and Fisher’s exact T-test were used for significance testing (*p < 0,05, **p < 0,01, ***p < 0,001). FDR correction was used for multiple testing correction. Mutation carrier subgroups were compared to non-carriers. NC = non-carriers, SMC = symptomatic mutation carriers, PMC = presymptomatic mutation carriers. CDR = Clinical Dementia Rating scale, MMSE = Mini Mental State Examination test

### Cross-sectional Aβ analysis

**Fig. 1:**
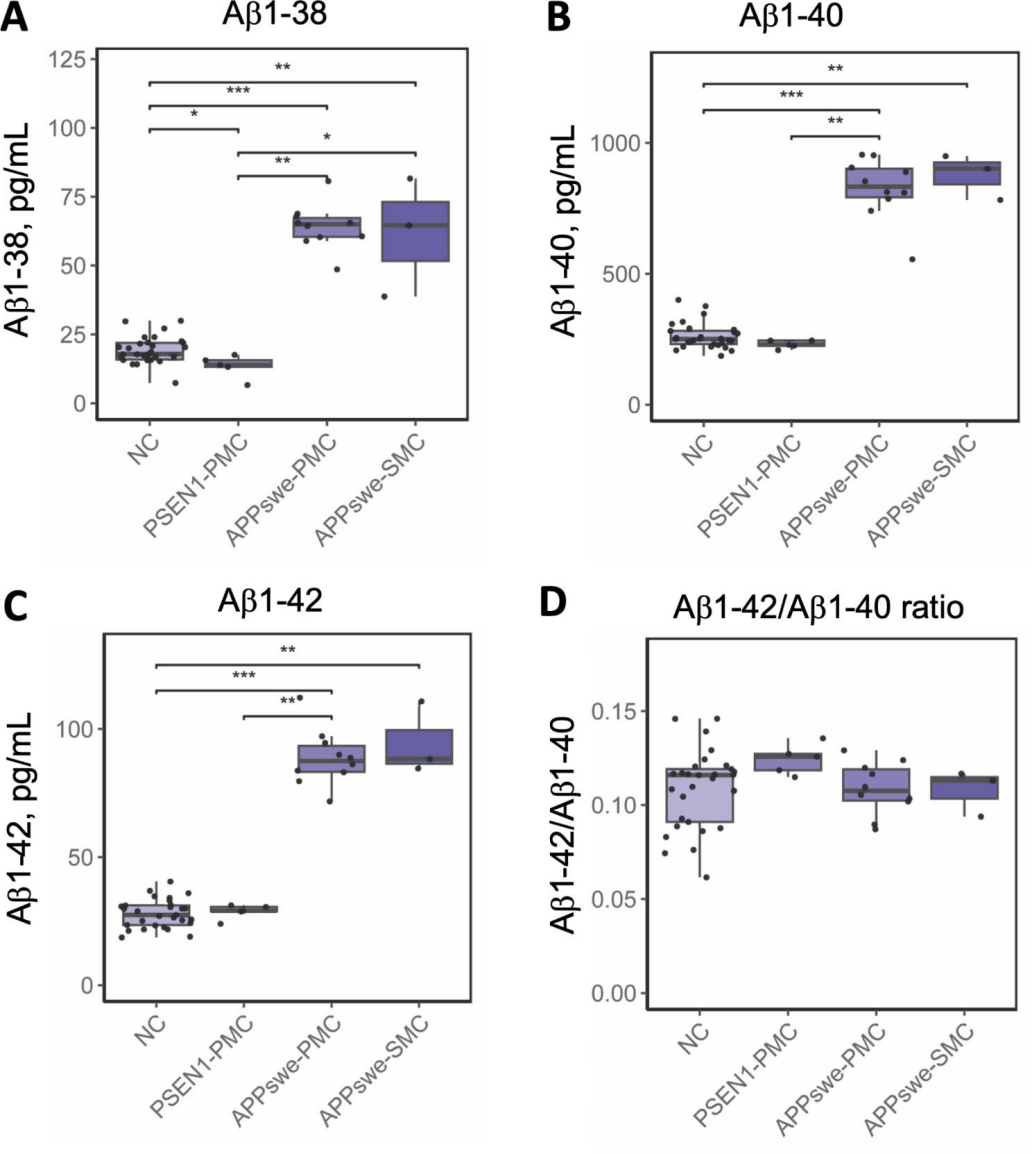
*Plasma concentrations of Aβ isoforms at baseline.* Cross-sectional baseline data from *APP*swe and *PSEN1* (p.H163Y). Plasma Aβ1–38, Aβ1–40 and Aβ1–42 concentrations were more than 3-fold increased in *APP*swe PMC (*n* = 10) and SMC (*n* = 3) compared to NC (*n* = 29) (Kruskal Wallis *p* < 0.001). In *PSEN1* (p.H163Y) only the Aβ1–38 concentrations were significantly decreased in PMC (*n* = 5) compared to NC (*n* = 29) after FDR correction for multiple testing (*p < 0.05, **p < 0.01, ***p < 0.001). NC = non-carriers, PMC = presymptomatic mutation carriers, SMC = symptomatic mutation carriers

Median plasma Aβ1–38, Aβ1–40 and Aβ1–42 concentrations were increased more than 3-fold in *APP*swe PMC and SMC (*n* = 13) compared to NC (*n* = 29) (Mann-Whitney *U*, *p* < 0.01) (Fig. 1; Table 1). In contrast, the plasma Aβ1–42/1–40 ratios were similar in SMC and PMC compared to NC. There were no differences in any of the Aβ peptide concentrations between *APP*swe PMC and SMC.

At baseline, *PSEN1* (p.H163Y) PMC (*n* = 5), showed a 22% reduction in median plasma Aβ1–38 concentrations compared to NC (*n* = 29) (Mann-Whitney *U*, *p* = 0.01, Fig. 1; Table 1). Furthermore, the plasma Aβ1–38, Aβ1–40 and Aβ1–42 levels in *PSEN1* PMC were lower compared to *APP*swe PMC and SMC (Fig. 1).

### Repeated-measures Aβ analysis

**Fig. 2:**
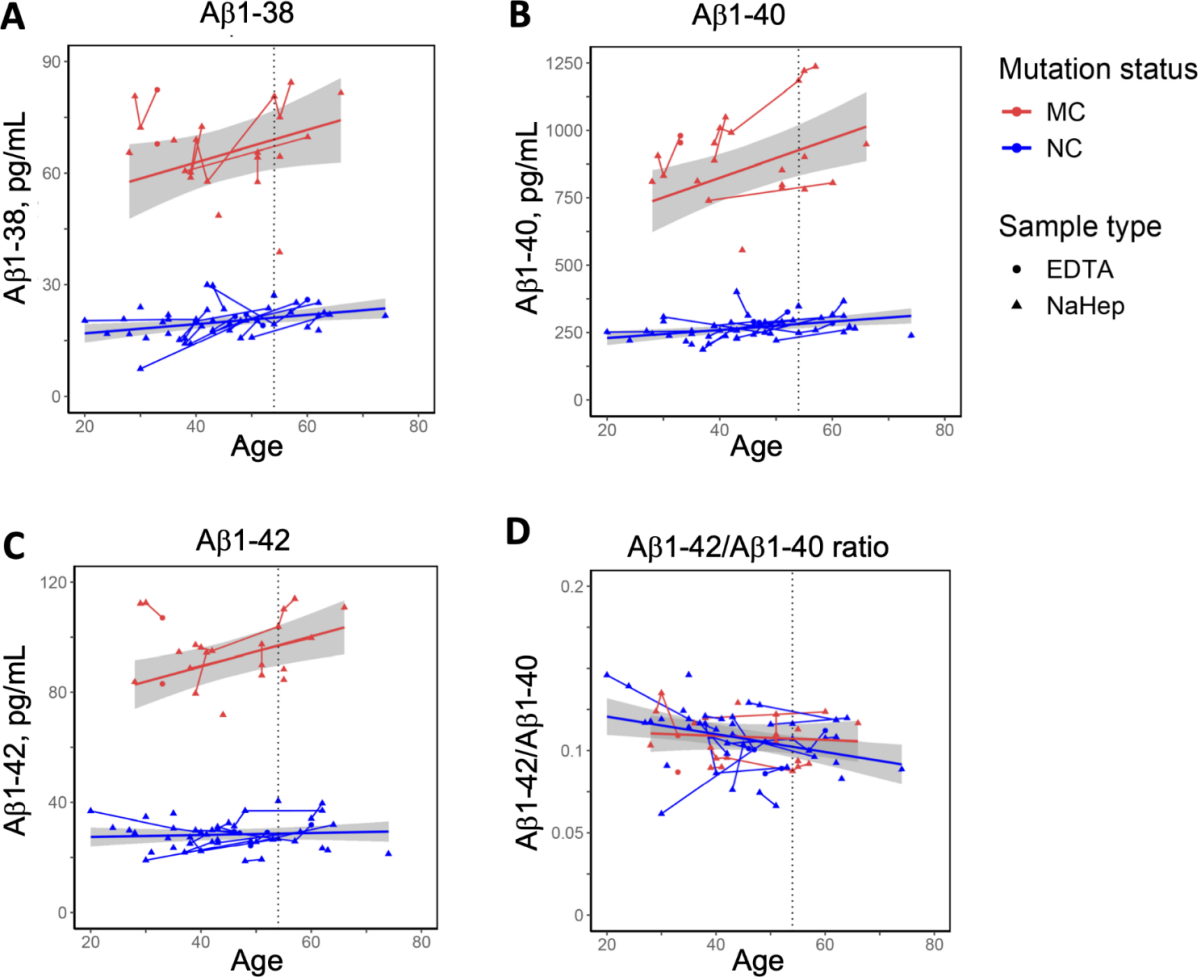
*Plasma concentrations of Aβ isoforms in APPswe*,* repeated-measures*. Plasma concentrations from repeated-measures of **(A)** Aβ1–38, **(B)** Aβ1–40, **(C)** Aβ1–42 and **(D)** Aβ1–42/ 1–40 ratio. Trajectories indicating fitting of mixed-effects data with confidence bands for MC (23 samples) and NC (49 samples) at the group level, as well as repeated-measures at the individual level. Two individuals had symptom onset during follow-up. The dotted line at 54 years of age represents the mean age at onset in the *APP*swe family. NC = Non-carriers, MC = Mutation carriers

Estimates in the mixed-effects model of *APP*swe MC and NC controls indicated highly increased levels of Aβ1–38, Aβ1–40 and Aβ1–42 in MC compared with NC (Table 2; Fig. 2), visualized in plots as a complete separation of the MC and NC plasma Aβ confidence bands at all ages (Fig. 2), in analogy with the cross-sectional baseline results. Furthermore, the results showed a relative increase of Aβ1–40 and Aβ1–42 levels with increasing age in *APP*swe MC compared to NC, as detected by the interaction term “mutation status*age” (Table 2). The Aβ1–42/1–40 ratio decreased with age and was not affected by mutation status.

**Table 2:** Mixed-effects models of plasma Aβ isoforms in APPswe MC vs. NC

	Age	Mut status	Mut status*Age
	Estimate [SE]	Estimate [SE]	Estimate [SE]
Aβ1–38 (pg/mL)	ns	46.5 [2.76]***	ns
Aβ1–40 (pg/mL)	ns	639 [34.4]***	4.95 [1.79]**
Aβ1–42 (pg/mL)	ns	67.9 [3.03]***	0.476 [0.151]**
Aβ1–42/1–40	-519*10^(-4) [184*10^(-4)]**	ns	ns

Mixed-effects models of repeated-measures data in LC-MS/MS analysis. 72 plasma samples were included from *APP*swe MC (*n* = 13, 23 samples) and NC (*n* = 29, 49 samples). Table showing estimates, the standard error (SE) within brackets and statistical significance (*p < 0.05, **p < 0.01, ***p < 0.001). Mut status = Mutation status, here mutation carriers compared to non-carriers. Age was centered to 54 years, the mean age at onset in the *APP*swe family. All models were corrected for *APOE* ɛ4 status and sex

In contrast, in the mixed-effects model of *PSEN1* (p.H163Y) and NC controls, presence of the mutation did not significantly affect the levels of Aβ peptides (Table 3), incongruent with the cross-sectional analysis which showed a mutation effect on Aβ1–38 levels (Table 1). However, there was an increase of Aβ1–38 and Aβ1–40, as well as a decrease of the Aβ1–42/1–40 ratio, with older age that was not affected by mutation status (Table 3). Noteworthy, the *PSEN1* (p.H163Y) and NC model showed some heteroskedasticity of residuals due to outliers (> 1.5IQR + 3Q). Explorative post hoc analysis of the *PSEN1* (p.H163Y) and NC model, applying robust standard errors, showed unchanged findings of an age effect on plasma Aβ1–38 (*p* = 0.005), Aβ1–40 (*p* = 0.022) and Aβ1–42/1–40 ratio (*p* = 0.049) (data not shown). Additionally, it provided evidence for a statistically significant reduction of Aβ1–38 levels in *PSEN1* MC compared to NC also in the longitudinal analysis (estimate − 2.739, SE 0.886, *p* = 0.026, data not shown).

**Table 3:** Mixed-effects models of plasma Aβ isoforms in PSEN1 (p.H163Y) MC vs. NC

	Age	Mut status	Mut status*Age
	Estimate [SE]	Estimate [SE]	Estimate [SE]
Aβ1–38 (pg/mL)	0.134 [0.044]**	ns	ns
Aβ1–40 (pg/mL)	1.44[0.434]**	ns	ns
Aβ1–42 (pg/mL)	ns	ns	ns
Aβ1–42/1–40	-507*10^(-4) [188*10^(-4)]**	ns	ns

Mixed-effects models of repeated-measures data in LC-MS/MS analysis. 69 plasma samples were included from *PSEN1* (p.H163Y) MC (*n* = 5, 20 samples) and NC (*n* = 29, 49 samples). Table showing estimates, the standard error (SE) within brackets and statistical significance (*p < 0.05, **p < 0.01, ***p < 0.001). Mut status = Mutation status, here mutation carriers compared to non-carriers. Age was centered to 52 years, the mean age at onset in the *PSEN1* family. All models were corrected for *APOE* ɛ4 status and sex

Sex and *APOE* ɛ4 status did not affect any of the biomarker trajectories. Anticoagulant additive was annotated in repeated-measures plots (Fig. 2, Suppl. Figure 2).

Exploratory mixed-effects models of *APP*swe MC, *PSEN1* (p.H163Y) MC and NC separately indicated a decrease of Aβ1–42/1–40 ratio with age in *PSEN1* MC and NC, but not in *APP*swe MC (Suppl. Table 1). In NC and *APP*swe MC there was an increase of Aβ1–38 and Aβ1–40 with older age, as well as an increase of Aβ1–42 in *APP*swe MC, but none of these changes could be observed in *PSEN1* MC alone (Suppl. Table 1).

### Associations between CSF and plasma Aβ concentrations

Previously analyzed CSF Aβ peptide concentrations (Aβ38, Aβ40, Aβ42 and Aβ42/40 ratio), T-tau and P-tau181 [28, 29, 40, 41] were available from the same plasma sampling dates in a subset of individuals (cross-sectionally 15 to 19 sampling occasions, longitudinally 23 to 27 samples). Supplementary Fig. 3 illustrates the scatterplots of each of the plasma Aβ isoform concentrations and the respective concentrations in the CSF. Mixed-effects models did not indicate any association between plasma Aβ peptide concentrations and the CSF biomarkers (CSF Aβ38, Aβ40, Aβ42, Aβ42/40, T-tau and P-tau181) (data not shown). Nor could any correlation be detected in an exploratory Spearman analysis (15 to 19 samples, including both MC and NC), although with a very limited sample size (data not shown).

## Discussion

We found variant-specific effects on plasma Aβ peptide levels in a longitudinal cohort of two families with autosomal dominant Alzheimer disease (ADAD), including pronounced elevations of Aβ peptides in *APP*swe MC. Furthermore, there was no change in the Aβ1–42/1–40 ratio in *APP*swe MC with aging, which may suggest a stable ratio over life. Several plasma Aβ peptide concentrations however increased with age in both *APP*swe MC and NC, suggestive of indirect effects on the Aβ ratio that are not related to a decrease of Aβ1–42. Lastly, the data did not support any association between plasma Aβ peptide and CSF biomarker concentrations in a subset with *APP*swe MC, *PSEN1* (p.H163Y) MC and NC. These in vivo findings raise concerns about the utility of plasma Aβ in predicting level of brain amyloid pathology, and caution must be applied in individuals at-risk for ADAD.

Most strikingly, all plasma Aβ peptide levels (Aβ1–38, Aβ1–40 and Aβ1–42) were markedly (3-fold) increased in *APP*swe MC compared to NC controls. Plasma Aβ levels in MC were completely separated from the levels in NC in both cross-sectional and repeated-measures analyses in all studied age groups (range 20 to 83). In contrast, there was no difference in the Aβ1–42/1–40 ratio between *APP*swe MC and controls in any of the comparisons. The biological effect of the *APP*swe variant, with an increased affinity to and N-terminal cleavage by BACE1, is known to result in an over-production of Aβ1-x peptides [42, 43]. Thus, these findings agree with early exploratory in vivo analyses of human *APP*swe fibroblast cultures and plasma concentrations showing a 2- to 3-fold increase of Aβ peptide [5, 36], but indicate lower concentrations of Aβ peptides than in vitro findings (4- to 8-fold increase) from experiments with transfected cell lines [4, 7]. In vitro studies of the *PSEN1* (p.H163Y) variant detected increased production of Aβ42 [44] or both Aβ42 and Aβ40 combined, resulting in an increased Aβ42/Aβ40 ratio [45]. The previously reported increase in Aβ42/Aβ40 ratio was not replicated in plasma in the current repeated-measures analysis. However, plasma Aβ results showed lower levels of plasma Aβ1–38 in *PSEN1* (p.H163Y) MC compared to non-carriers both cross-sectionally and longitudinally, when applying robust standard errors. Such observations of reduced concentrations of plasma Aβ38 in *PSEN1* MC compared to controls have previously been connected to an impaired enzymatic function in the physiologic last step of C-terminal cleavage of Aβ, impeding conversion of Aβ42 to Aβ38 in *PSEN1* variants [46]. The variations in Aβ peptide concentrations between in vitro and in vivo experiments of ADAD genetic variants could be influenced by multiple factors (preanalytical factors, model, matrix, assay etc.), thus making direct comparisons difficult, and should be further addressed.

Evaluation of temporal dynamics indicated increased plasma Aβ concentrations in both *APP*swe MC and NC with aging. Repeated measurements supported an increase in all Aβ peptides in *APP*swe MC over time and, although this effect was very small in comparison to the intraindividual variability in the data, the increase of both Aβ1–40 and Aβ1–42 is likely to have contributed to a rather stable Aβ1–42/1–40 ratio with aging. When comparing symptomatic and presymptomatic *APP*swe MC at baseline, no differences could be detected in any of the Aβ peptides, possibly due to very small subgroups. In NC alone, there was an increase of Aβ1–40 with aging that more clearly contributed to the decrease of the Aβ ratio in the context of a relatively unchanged Aβ1–42. *PSEN1* MC did not clearly deviate from NC controls and a decrease of the Aβ1–42/1–40 ratio was the only significant change when analyzing repeated-measures in *PSEN1* MC exclusively. An early descriptive report suggested that, although generally elevated, plasma Aβ40 remained unchanged and Aβ42 slightly increased in symptomatic compared to presymptomatic *APP*swe MC. Also, *PSEN1* pathogenic variants caused increased levels of plasma Aβ42 that remained unchanged in symptomatic compared to presymptomatic individuals [5]. A larger and more recent cross-sectional evaluation of plasma Aβ in a British ADAD cohort showed that symptomatic carriers of variants in *PSEN1*, but not *APP*, had higher Aβ42/40 ratios than presymptomatic carriers, but neither *APP* nor *PSEN1* plasma Aβ42/40 ratios were associated with estimated years to symptom onset [46]. Down syndrome, with three alleles of *APP* and a resulting over-production of Aβ product, represents another at-risk group for genetic AD. Unlike older conflicting evidence, more recent assessments of Down syndrome plasma Aβ levels indicate indistinguishable Aβ42 concentrations and Aβ42/40 ratios both in individuals with and without AD dementia [47–50]. Hence, our data, showing a small increase in plasma Aβ1–42, but no change in Aβ1–42/1–40 ratio in *APP*swe MC over time, are consistent with the previously reported findings of unchanged or higher plasma Aβ42 and Aβ42/40 ratio in symptomatic individuals in Down syndrome and ADAD cohorts [46–50]. The decrease in Aβ1–42/1–40 ratio with aging in *PSEN1* (p.H163Y) mutation carriers however instead rather imitates the change in NC in our data and align to the decrease in plasma Aβ42/40 previously described in sporadic AD [18–20], underlining the influence of mutation-specific effects on Aβ processing and turnover [46].

Interestingly, in our cohort plasma Aβ peptides or the Aβ1–42/1–40 ratio did not associate to any of the core CSF AD biomarkers, in contrast to the previously reported positive association between plasma P-tau181 and CSF tau biomarkers in the same cohort [29]. In sporadic AD, it has been emphasized that the plasma Aβ42/40 ratio is only marginally changed (around a 10% reduction in amyloid PET positive AD cases as compared with controls) [24], which makes comparisons to CSF challenging. Also, blood is a complex matrix and concentrations of plasma Aβ peptides can be affected by factors such as microenvironment, tissue-specific expression of proteins relevant for Aβ production and turn-over and presence of Aβ-binding proteins and cells [51]. A previous study in the current cohort showed that concentrations of other plasma biomarkers such as P-tau181, neurofilament light chain (NfL) and glial fibrillary acidic protein (GFAP) were elevated in MC, with increases detected already in the presymptomatic phase [29]. This pattern was repeatedly reported for blood-based P-tau, NfL and GFAP in various ADAD cohorts [29, 52–56] and early changes in plasma GFAP have been suggested to reflect Aβ related astrocytic reactivity [29, 56–58]. Furthermore, the small subset of *APP*swe MC, with large elevations of plasma Aβ peptides, had CSF Aβ peptide concentrations similar to NC and *PSEN1* (p.H163Y) MC. The observation that *APP*swe MC [40, 59] and other genetic groups with Aβ over-production, such as individuals with *APP* duplications and Down syndrome [48, 60, 61], do not appear to have increased Aβ concentrations in CSF has been reproducibly shown but is, to our knowledge, still unexplained. If there was a true association between plasma Aβ peptides and CSF amyloid and tau biomarkers in the current dataset it might have gone undetected due to low power in this small subset. However, we still suggest that other plasma biomarkers, reflecting amyloid and tau pathology in the CNS, that are less sensitive to gene variants in *APP* and *PSEN1* will outperform plasma Aβ42 and the Aβ42/40 ratio both at the individual and at the group level. The current findings of unselective increases of plasma Aβ peptides in *APP*swe MC do not support that they are significantly affected by brain plaque formation or decreasing levels of Aβ42 in CSF. These changes in *APP*swe MC, and other genetic variants, are likely affected by a ubiquitous peripheral production in different cell types throughout the body (i.e. skin fibroblasts, skeletal muscle cells, platelets etc.). Altogether, we hypothesize that the performance of plasma Aβ biomarkers will not allow for prediction of level of brain amyloid pathology or disease activity in ADAD.

### Limitations

Our findings are exploratory and the main limitation is the small sample size, as a consequence of the rarity of Swedish ADAD cases. Next, reliable measurement of the 10-fold lower concentration of plasma Aβ compared to the levels in CSF remains a challenge even for more sensitive immunoassays and modern mass spectrometry-based methods [22]. The correlation between plasma and CSF levels of Aβ could be affected by the use of different assays (MS versus electrochemiluminescence based methodology) for quantification, with varying specificity for full-length Aβ peptides, as well as the time between measurement of plasma and CSF concentrations. Performance of plasma Aβ assays might be influenced by preanalytical confounders such as number of freeze-thaw cycles, time to centrifugation and storage, diurnal effects and variation caused by choice of anticoagulant in the collection tube. Collection of blood in our cohort was extended over more than two decades, which introduces an increased risk of variation in preanalytical handling, as has been addressed elsewhere [29]. Furthermore, minor diurnal effects may have been introduced by venipuncture in non-fasting patients during varying times of the day. However, time to centrifugation and storage has to our knowledge been compliant to current recommendations. A modernization of in-house standard operating procedures in 2015 included a switch from sodium heparin to EDTA tube anticoagulant and both anticoagulants were allowed for in the current data, which is another limitation of the study. Several studies of preanalytical procedures have indicated that Aβ40 and Aβ42 concentrations are higher in sodium or lithium heparin compared to K2/K3 EDTA collection tubes [62–65]. However, this difference between collection tubes was not replicated when evaluating only mass spectrometry-based assays, supporting that MS Aβ measurements might be less affected by different anticoagulants than other blood-based biomarkers [65]. We could not detect lower Aβ concentrations in EDTA collection tubes at the group level and all samples were included in the analyses regardless of collection tube additive.

## Conclusion

In conclusion, our results further support that ADAD genetic variants have heterogeneous plasma Aβ profiles, which should make us cautious when making interpretations in unselected clinical cohorts. It is clear that plasma Aβ concentrations in *APP*swe mutation carriers indirectly reflect the genetic status in these at-risk individuals and can unintentionally disclose the genetic status in presymptomatic individuals. Furthermore, the Aβ ratio was not associated to age or clinical status in *APP*swe mutation carriers. Together, the current findings indicate that plasma Aβ biomarkers might not be well suited for use in clinical trials or for screening purposes in clinical practice in individuals at-risk of developing ADAD.

## Electronic supplementary material

Below is the link to the electronic supplementary material.

Supplementary Material 1

## Data Availability

The data underlying these results are not publicly available due to the need to maintain confidentiality of sensitive genetic information in this small cohort. Anonymized data may however be shared upon reasonable request and if in agreement with EU legislation on the general data protection regulation, the Swedish Ethical Review Authority and fulfilling appropriate routines of data sharing.

## Abbreviations

Aβ: Amyloid beta
ADAD: Autosomal dominant Alzheimer disease
APP: Amyloid precursor protein
EDTA: Ethylenediaminetetraacetic acid
FDR: False discovery rate
MC: Mutation carriers
NC: Non-carriers
PMC: Presymptomatic mutation carriers
PSEN1: Presenilin 1
PSEN2: Presenilin 2
SMC: Symptomatic mutation carriers
