# Supplementary material for: Gene-variant specific effects of plasma amyloid-β levels in Swedish autosomal dominant Alzheimer disease
Source: Alzheimers Res Ther. 2024 Sep 25;16:207. doi: 10.1186/s13195-024-01574-w (PMC11423518; doi:10.1186/s13195-024-01574-w)
Supplement: Supplementary file 1 — Supplementary Material 1 [file 13195_2024_1574_MOESM1_ESM.docx]

Supplementary Material

Johansson C, Thordardottir S, Laffita-Mesa J, *et al.* Gene-variant specific effects of plasma amyloid-β levels in Swedish autosomal dominant Alzheimer disease

**Corresponding author:** Caroline Graff, Karolinska Institutet, Dept NVS, Division of Neurogeriatrics, Center for Alzheimer Research, Bioclinicum J10:20, Visionsgatan 4, 171 64 Solna, Sweden.

**Tel:** +46 (0) 73 383 93 99, **E-mail**: caroline.graff@ki.se

Table of contents

Supplementary Tables

**Supplementary Table 1.** Exploratory mixed-effects models of plasma Aβ isoforms

Supplementary Figures

**Supplementary Fig. 1.** Flow chart of sample cohort

**Supplementary Fig. 2.** Plasma concentrations of Aβ isoforms in *PSEN1* p.H163Y, longitudinal data

**Supplementary Fig. 3.** Plasma Aβ versus CSF Aβ isoform concentrations

Supplementary Table 1

| *Exploratory mixed-effects models of plasma Aβ isoforms in APPswe MC* | | | |
| --- | --- | --- | --- |
|  | Age | APOE4+ | Sex |
|  | Estimate [SE] | Estimate [SE] | Estimate [SE] |
| Aβ1-38 (pg/mL) | 0.509 [0.229]* | ns | ns |
| Aβ1-40 (pg/mL) | 7.850 [2.407]** | ns | ns |
| Aβ1-42 (pg/mL) | 0.581 [0.186]** | ns | ns |
| Aβ1-42/1-40 (ratio) | ns | ns | ns |
| Exploratory mixed-effects models of repeated-measures data in LC-MS/MS analysis. 23 plasma samples were included from *APP*swe MC (n=13). The estimates of predictors indicate the average change in the dependent variable with a 1-unit increase of the predictor for the *APP*swe study sample, i.e. the average change in peptide concentration per year for the “Age” predictor. Table showing estimates, the standard error (SE) within brackets and statistical significance (*p<0.05, **p<0.01, ***p<0.001). | | | |

| *Exploratory mixed-effects models of plasma Aβ isoforms in PSEN1 p.H163Y MC* | | | |
| --- | --- | --- | --- |
|  | Age | APOE4+ | Sex |
|  | Estimate [SE] | Estimate [SE] | Estimate [SE] |
| Aβ1-38 (pg/mL) | ns | ns | ns |
| Aβ1-40 (pg/mL) | ns | ns | ns |
| Aβ1-42 (pg/mL) | ns | ns | ns |
| Aβ1-42/1-40 (ratio) | -1.097*10^(-3) [0.293*10^(-3)]** | ns | ns |
| Exploratory mixed-effects models of repeated-measures data in LC-MS/MS analysis. 20 plasma samples were included from *PSEN1* p.H163Y MC (n=5). The estimates of predictors indicate the average change in the dependent variable with a 1-unit increase of the predictor for the *PSEN1* p.H163Y study sample, i.e. the average change in peptide ratio per year for the “Age” predictor. Table showing estimates, the standard error (SE) within brackets and statistical significance (*p<0.05, **p<0.01, ***p<0.001). | | | |

| *Exploratory mixed-effects models of plasma Aβ isoforms in NC controls* | | | |
| --- | --- | --- | --- |
|  | Age | APOE4+ | Sex |
|  | Estimate [SE] | Estimate [SE] | Estimate [SE] |
| Aβ1-38 (pg/mL) | 0.132 [0.046]** | ns | ns |
| Aβ1-40 (pg/mL) | 1.435 [0.479]** | ns | ns |
| Aβ1-42 (pg/mL) | ns | ns | ns |
| Aβ1-42/1-40 (ratio) | -0.516*10^(-3) [0.199*10^(-3)]* | ns | ns |
| Exploratory mixed-effects models of repeated-measures data in LC-MS/MS analysis. 49 plasma samples were included from non-carriers (n=29). The estimates of predictors indicate the average change in the dependent variable with a 1-unit increase of the predictor for the NC study sample, i.e. the average change in peptide concentration per year for the “Age” predictor. Table showing estimates, the standard error (SE) within brackets and statistical significance (*p<0.05, **p<0.01, ***p<0.001). | | | |

Supplementary Fig. 1

Flow chart of sample cohort


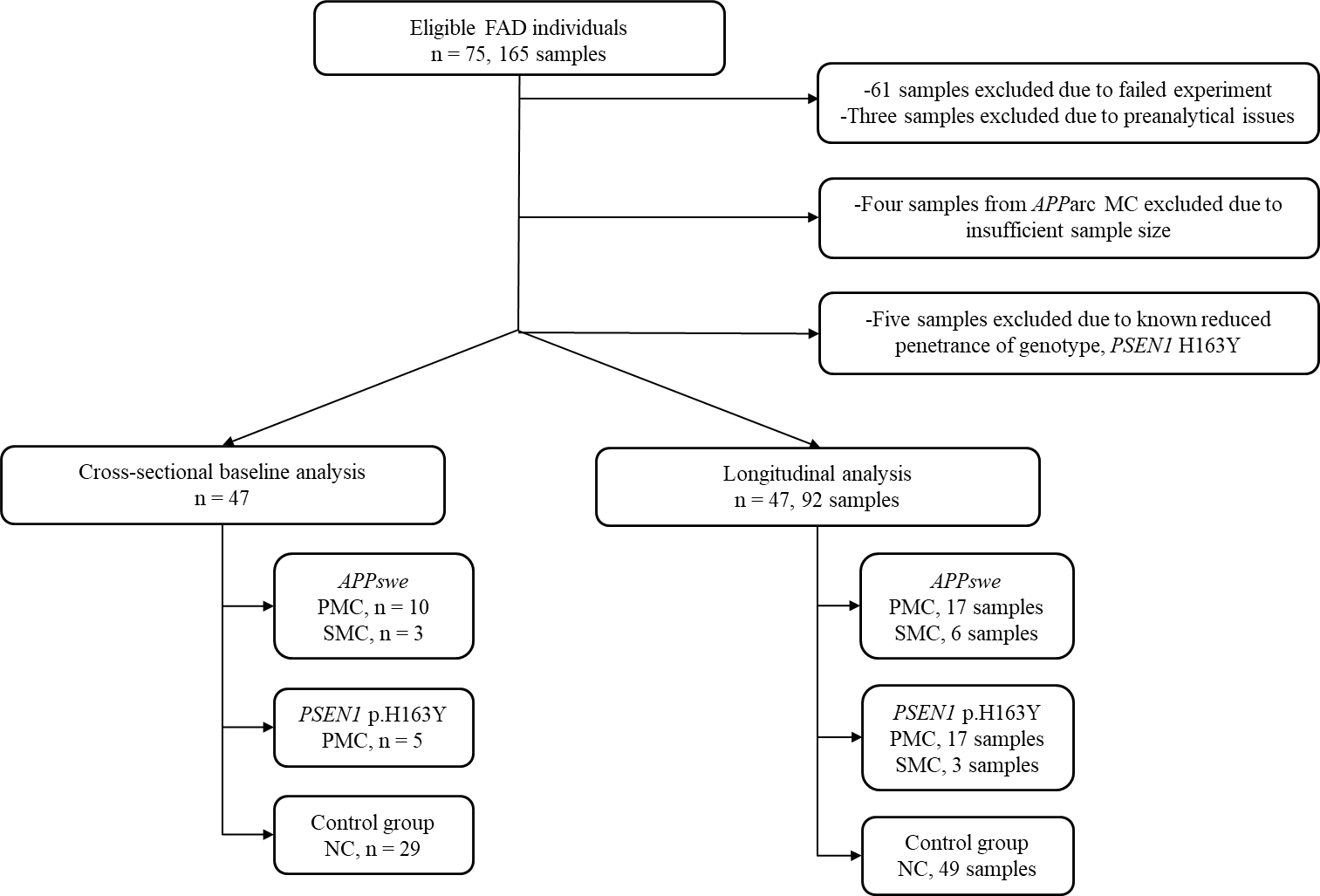


Flow chart of the sample cohort selection. 165 plasma samples from 75 individuals were eligible for inclusion. In total, cross-sectional analysis included 47 samples and repeated-measures analysis included 92 samples from 47 individuals. Explanation for excluded samples: 61 samples (31 *APP*arc MC, 3 *PSEN1* p.H163Y MC and 27 NC) from 23 individuals were removed due to technical issues in one experimental run. Troubleshooting indicated low signals affecting the lower part of the calibration curve, with a median less than 1.5*LLOQ for plasma Aβ1-42 in *APP*arc MC, *PSEN1* MC and NC in this experimental run. Four *APP*arc MC samples (corresponding to n=3 individuals) from another experimental run were excluded due to insufficient sample size. Three additional samples were removed that showed indication of preanalytical issues (failed injection, one sample with undetectable Aβ1-42 and one sample with extreme outlier values in another biomarker experiment), whereof one individual was completely removed from analysis. Last, five samples from a *PSEN1* p.H163Y MC with known reduced penetrance were excluded. NC = Non-carriers, PMC = Presymptomatic mutation carriers, SMC = Symptomatic mutation carriers.

Supplementary Fig. 2

Plasma concentrations of Aβ isoforms in *PSEN1* p.H163Y, repeated-measures


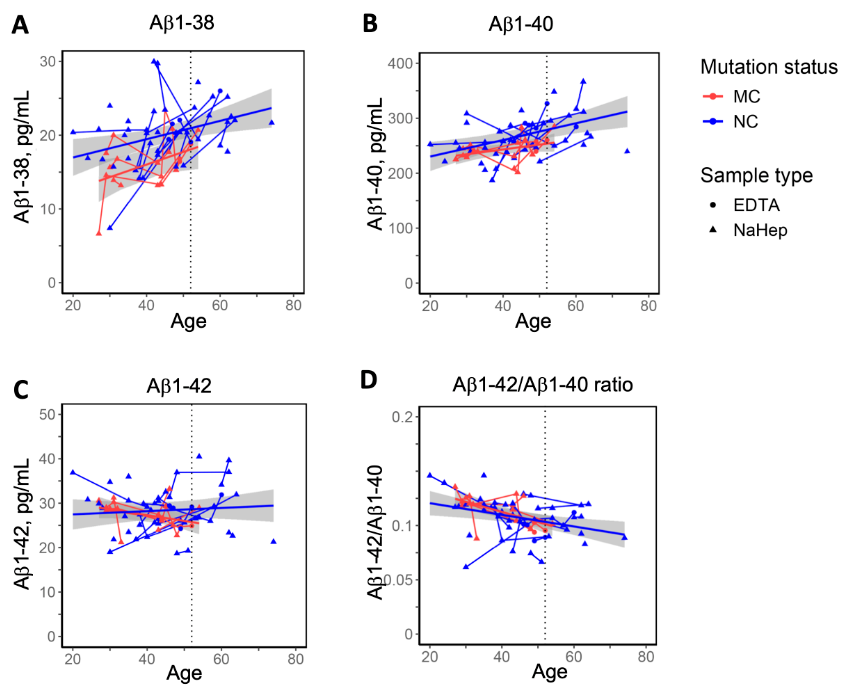


Plasma concentrations from repeated-measures of **(A)** Aβ1-38, **(B)** Aβ1-40, **(C)** Aβ1-42 and **(D)** Aβ1-42/ 1-40 ratio. Trajectories indicating fitting of mixed-effects data with confidence bands for MC (20 samples) and NC (49 samples) at the group level, as well as repeated measures at the individual level. One individual converted during follow-up. Dotted line at 52 years of age represents the mean age at onset in the *PSEN1* p.H163Y family. NC = Non-carriers, MC = Mutation carriers.

Supplementary Fig. 3

Plasma Aβ versus CSF Aβ isoform concentrations


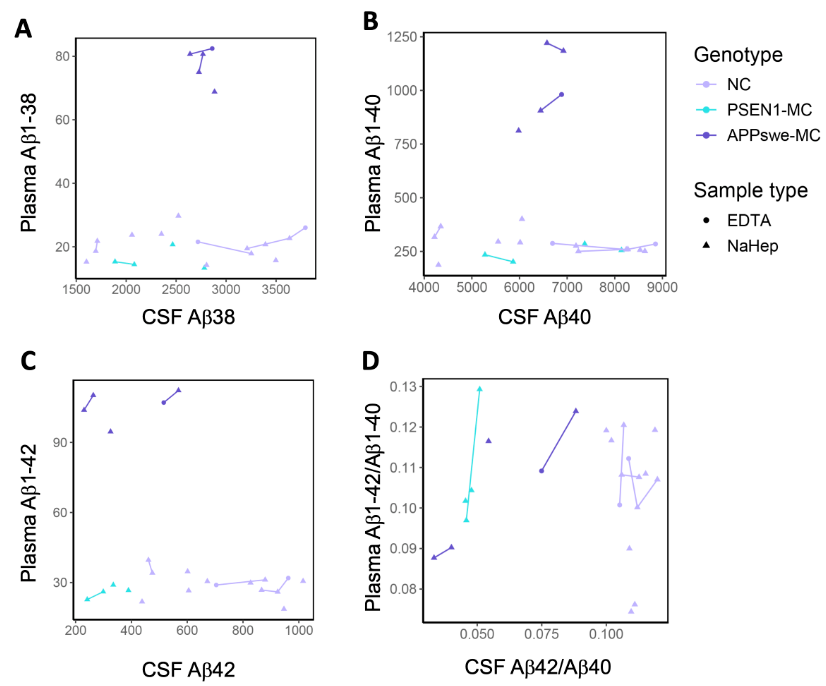


Plasma versus CSF concentrations of **(A)** Aβ1-38, **(B)** Aβ1-40, **(C)** Aβ1-42 and **(D)** Aβ1-42/ 1-40 ratio in a subset of the cohort (14 NC and 9 MC samples). Trajectories indicate repeated measures at the individual level. Linear mixed models (23 samples) and Spearman correlation (15 baseline samples) indicated no association between plasma and CSF concentrations.
